# High expression of S100A2 predicts poor prognosis in patients with endometrial carcinoma

**DOI:** 10.1186/s12885-022-09180-5

**Published:** 2022-01-18

**Authors:** Qinzhen Zhang, Tianxiang Xia, Chenxiang Qi, Jun Du, Chunping Ye

**Affiliations:** 1 The First Clinical Medical College, Nanjing Medical University, 211166 Nanjing, Jiangsu China; 2 Department of Physiology, Nanjing Medical University, 101 Longmian Avenue, Jiangning District, 211166 Nanjing, Jiangsu China; 3 Department of Obstetrics and Gynecology, Nanjing Maternity and Child Health Care Hospital, Women’s Hospital of Nanjing Medical University, 123 Mochou Road, 210004 Nanjing, Jiangsu China

**Keywords:** S100A2, Overall survival, Prognosis, Endometrial carcinoma, Bioinformatics

## Abstract

**Background:**

S100A2, a member of the S100 protein family, is abnormally expressed and plays a vital role in multiple cancers. However, little is known about the clinical significance of S100A2 in endometrial carcinoma.

**Methods:**

Clinicopathological data were obtained from The Cancer Genome Atlas (TCGA), Genotype-Tissue Expression (GTEx), Gene Expression Omnibus (GEO), and Clinical Proteomic Tumor Analysis Consortium (CPTAC). First, the expression and prognostic value of different S100 family members in endometrial carcinoma were evaluated. Subsequently, the Kaplan–Meier plotter and Cox regression analysis were used to assess the prognostic significance of S100A2, while the association between S100A2 expression and clinical characteristics in endometrial carcinoma was also analyzed using logistic regression. A receiver operating characteristic (ROC) curve and a nomogram were constructed. The putative underlying cellular mechanisms were explored using Kyoto Encyclopedia of Genes and Genomes (KEGG) pathway enrichment analysis and gene set enrichment analysis (GSEA).

**Results:**

Our results revealed that S100A2 expression was significantly higher in endometrial carcinoma tissue than in non-cancerous tissue at both the mRNA and protein levels. Analysis of Kaplan–Meier plotter data revealed that patients with high S100A2 expression had shorter overall survival (OS) and disease specific survival (DSS) compared with those of patients with low S100A2 expression. Multivariate Cox analysis further confirmed that high S100A2 expression was an independent risk factor for OS in patients with endometrial carcinoma. Other clinicopathologic features found to be related to worse prognosis in endometrial carcinoma included age, clinical stage, histologic grade, and tumor invasion. Importantly, ROC analysis also confirmed that S100A2 has a high diagnostic value in endometrial carcinoma. KEGG enrichment analysis and GSEA revealed that the estrogen and IL-17 signaling pathways were significantly upregulated in the high S100A2 expression group, in which estrogen response, JAK-STAT3, K-Ras, and TNFα/NF-κB were differentially enriched.

**Conclusions:**

S100A2 plays an important role in endometrial carcinoma progression and may represent an independent diagnostic and prognostic biomarker for endometrial carcinoma.

**Supplementary Information:**

The online version contains supplementary material available at 10.1186/s12885-022-09180-5.

## Background

Endometrial carcinoma is one of the most commonly diagnosed gynecological malignancies, with nearly 400,000 new cases reported in 2020 [1]. It comprises a group of malignant epithelial tumors that originate in the inner lining of the uterus and frequently occur in perimenopausal and postmenopausal women. The incidence of endometrial carcinoma has shown a gradual increase over recent years concomitant with an improvement in economic conditions and an increase in the average life expectancy. Despite the survival rate of patients with endometrial carcinoma seems optimistic [2], the validated biological markers for its detection remains poor.

The S100s encompass a family of calcium-binding proteins composed of more than 20 members that share a conserved structure comprising two EF-hand domains with a high affinity for calcium ions [3]. Additionally, S100A2 can also bind zinc, which significantly reduces its affinity for calcium [4, 5]. Upon binding of those ions, most S100 proteins undergo conformational changes, which allows them to interact with other proteins and thereby play key roles in a wide range of cellular functions. Although S100 family members share structural similarity, they serve different biological functions [6]. Most *S100* genes, including those forming the S100A group (*S100A1–14*, *S100A7A*, and *S100A16*), are located in human chromosome 1q21, a region frequently associated with recombination events in tumor tissues, resulting in the uncontrolled expression of S100 members, such as the upregulation of S100A11 and S100A14 reported in breast cancer [7]. Additionally, high expression levels of S100A2, S100A4, S100A6, S100A7A, S100A10, S100A14, S100A16, S100B, and S100P have been associated with lower survival, whereas the upregulated expression of S100A1, S100A13, S100A5, S100A13, and S100G proteins have been associated with improved survival in patients with ovarian cancer [8]. Dysregulated expression of S100 family members can impair the regulation of diverse cellular functions such as proliferation, apoptosis, migration, and differentiation of cancer cells [9, 10]. In colorectal cancer, S100A2 acts as a tumor promoter by modulating glycolytic reprogramming [11]. Nevertheless, how S100 proteins affect the pathogenesis and prognosis of endometrial carcinoma remains to be determined.

The aim of this study was to identify members of the S100 gene family with prognostic value in endometrial carcinoma using comprehensive bioinformatic analysis. First, we compared the mRNA expression pattern of each S100 member between carcinoma tissues and normal tissues and assessed the prognostic role of S100 mRNA expression in patients with endometrial carcinoma. Subsequently, we selected S100A2 from the S100 family members and evaluated the correlations between S100A2 expression and clinical characteristics in endometrial carcinoma patients, and sought to determine the biological pathways related to S100A2 using Gene Set Enrichment Analysis (GSEA) and Kyoto Encyclopedia of Genes and Genomes (KEGG) pathway enrichment analysis. Our results suggested that S100A2 may be a promising diagnostic and prognostic biomarker for endometrial carcinoma.

## Methods

### UALCAN

UALCAN is a comprehensive and interactive web resource that provides easy access to publicly available cancer OMICS data (The Cancer Genome Atlas (TCGA), MET500, and Clinical Proteomic Tumor Analysis Consortium (CPTAC) databases and allows users to identify biomarkers or perform *in silico* validation of potential genes of interest (http://ualcan.path.uab.edu/index.html). Here, the mRNA and protein expression of S100A2 in endometrial carcinoma was evaluated using TCGA, Human Protein Atlas, and CPTAC [12] databases.

### Gene Expression Profiling Interactive Analysis (GEPIA) dataset

GEPIA is a newly developed interactive web server for analyzing the RNA sequencing data from TCGA and Genotype-Tissue Expression (GTEx) projects (http://gepia.cancer-pku.cn/). GEPIA provides customizable functions such as tumor/normal differential expression analysis, profiling according to cancer types or pathological stages, and patient survival analysis, among others. The expression of S100A2 in endometrial carcinoma was analyzed in the GEPIA database [13].

### Gene-gene interaction and protein-protein interaction networks

GeneMANIA (https://genemania.org/) helps us predict the function of gene/gene sets and STRING (https://cn.string-db.org/) aims to predict associations between proteins, both of which were used to explore the S100A2 gene and protein network.

### Kaplan–Meier plotter database

The prognostic value of S100A2 in endometrial carcinoma was assessed according to overall survival (OS) and disease specific survival (DSS) using Kaplan–Meier plotter. Sources for the databases include Gene Expression Omnibus (GEO), The European Genome-phenome Archive (EGA), and TCGA (https://kmplot.com/analysis/). The primary purpose of the tool is the meta-analysis-based discovery and validation of survival biomarkers [14].

### Patients in TCGA database

*S100A2* gene expression in endometrial carcinoma and the corresponding clinical information data were downloaded from TCGA database (https://tcga-data.nci.nih.gov/tcga/) [15]. UCEC data were included the clinical stage, tumor grade, pathological subtypes, age and other data of patients. In this study, *S100A2* mRNA expression and its association with the OS of patients with endometrial carcinoma were also analyzed in TCGA-UCEC dataset. Based on the median mRNA expression values, patients with endometrial carcinoma were divided into high and low expression groups. Data were collected and analyzed using R3.6.3 software [16].

### Screening for integrated differentially expressed genes (DEGs)

The gene expression profiles (GSE 17025 and GSE 39099), based on the GPL570 platform, were obtained from the National Center for Biotechnology Information (NCBI) GEO database (https://www.ncbi.nlm.nih.gov/geo/). Adjusted *P*-values <0.05 and log2 FC >1 were set as cut-off criteria for screening out the upregulated DEGs. The list of significantly upregulated *S100* genes was exported separately.

### KEGG pathway enrichment analysis and GSEA for S100A2

In this study, an ordered list of genes based on the correlation between all genes and *S100A2* expression was generated using KEGG and GSEA. Data were collected and analyzed using R3.6.3 software [16]. KEGG is a collection of databases dealing with genomes, biological pathways, diseases, drugs, and chemical substances (www.kegg.jp/kegg/kegg1.html). Genes were determined to be differentially expressed based on an absolute fold change >1.5 and *P*_adj_ <0.05.

GSEA is a computational method that allows the determination of classes of genes or proteins that are overrepresented in a large set of genes or proteins and may have a statistically significant association with disease phenotypes [17]. The predefined gene set is from the MSigDB database (https://www.gsea-msigdb.org/gsea/msigdb/index.jsp). In this study, an ordered list of genes based on the correlation between all genes and *S100A2* expression was generated using GSEA. The enriched pathways were determined based on the nominal *P*-value and the normalized enrichment score (NES).

### Statistical analysis

The Pearson *χ*^2^ test was used to analyze the relationship between S100A2 expression and clinicopathological variables; Fisher’s exact test was used when needed. OS was defined as the time from random assignment until death due to any cause and DSS as the percentage of people in a study or treatment group who have not died from a specific disease in a defined period of time. Kaplan–Meier analysis was used to evaluate the survival of patients, and the log-rank test was used to test the significance. Univariate Cox proportional hazards regression was applied to estimate the individual hazard ratio (HR) for OS and DSS.

Cox proportional hazards regression was used to identify the independent prognostic factors that are significant for the prognosis of patients with endometrial carcinoma. SPSS 22.0 software was used for statistical analysis. The chi-square test was used to compare and analyze the clinical and pathological conditions of the high and low expression groups. The HR with 95% confidence interval (CI) was measured to estimate the hazard risk of individual factors. R language was used to draw the nomogram and build a prediction model. *P* < 0.05 indicates statistical significance, and *P* < 0.01 indicates highly statistical significance. All reported *P*-values were two-sided.

## Results

### Prognostic values of S100 family members in endometrial carcinoma

**Fig. 1:**
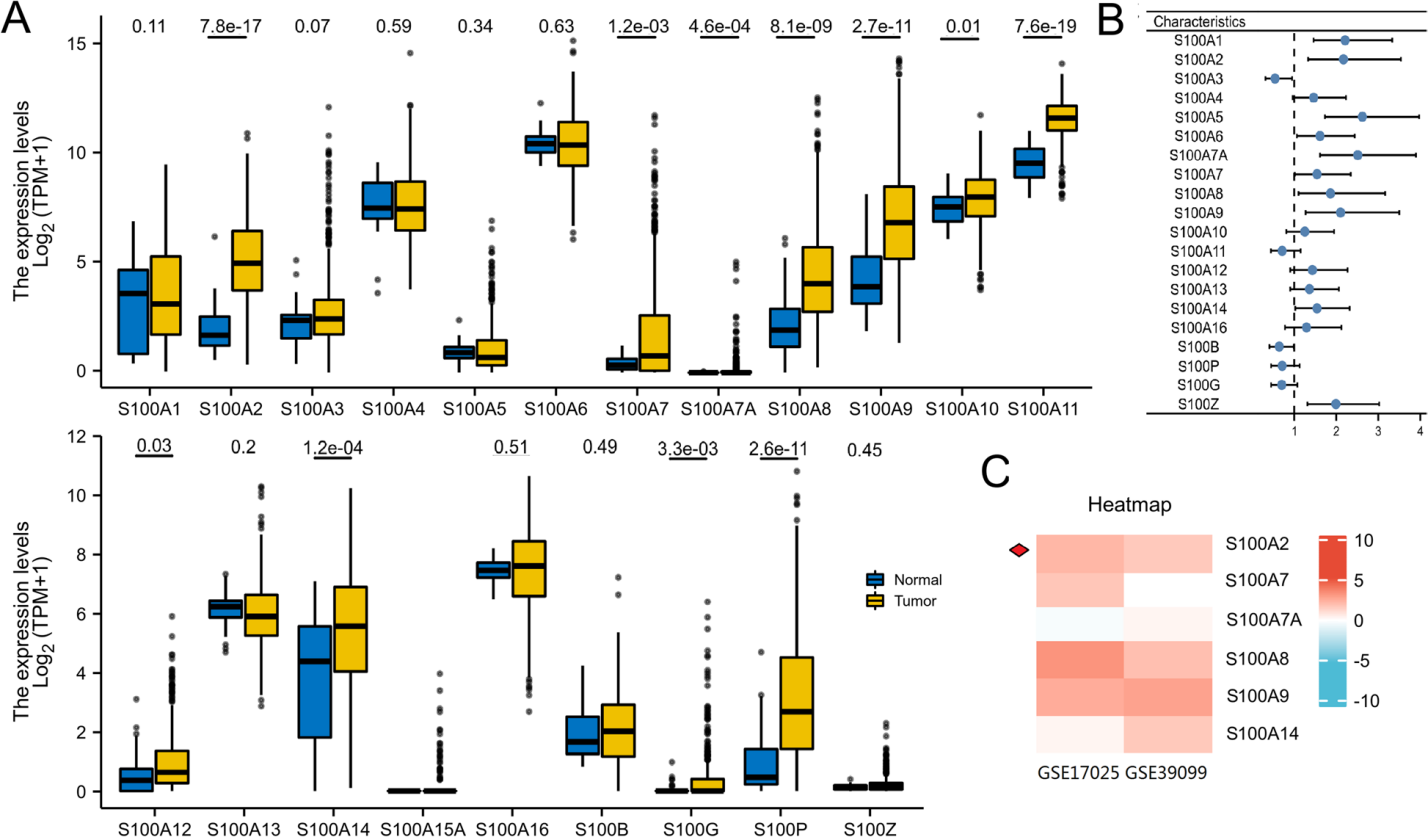
The prognostic value of S100 mRNA expression in endometrial cancer patients. (**a**) The mRNA expression of individual S100 members in endometrial carcinoma tissues. (**b**) Prognostic hazard ratios for individual S100 members in endometrial cancer patients. (**c**) Heatmap depicting the expression levels of individual S100 members

First, we evaluated the expression and prognostic role of each S100 family member in endometrial carcinoma. Among the 21 family members, 11 (*S100A2, S100A7, S100A7A, S100A8, S100A9, S100A10, S100A11, S100A12, S100A14, S100G* and *S100P*) were found to be significantly highly expressed in endometrial carcinoma tissue and 10 (*S100A1, S100A2, S100A5, S100A6, S100A7A, S100A7, S100A8, S100A9, S10014* and *S100Z*) were significantly correlated with OS in all patients with endometrial carcinoma (Fig. 1a, b). Six genes (*S100A2*, *S100A7*, *S100A7A*, *S100A8*, *S100A9*, and *S100A14*) were not only significantly highly expressed in endometrial carcinoma, but also associated with OS of endometrial carcinoma patients. To further explore the association between these selected genes and endometrial carcinoma, we downloaded two endometrial carcinoma gene expression profiles (GSE17025 and GSE39099) from the GEO database. The expressions of these six genes were depicted on a heatmap in Fig. 1c.

### High S100A2 expression is correlated with clinicopathologic features in patients with endometrial carcinoma

**Fig. 2:**
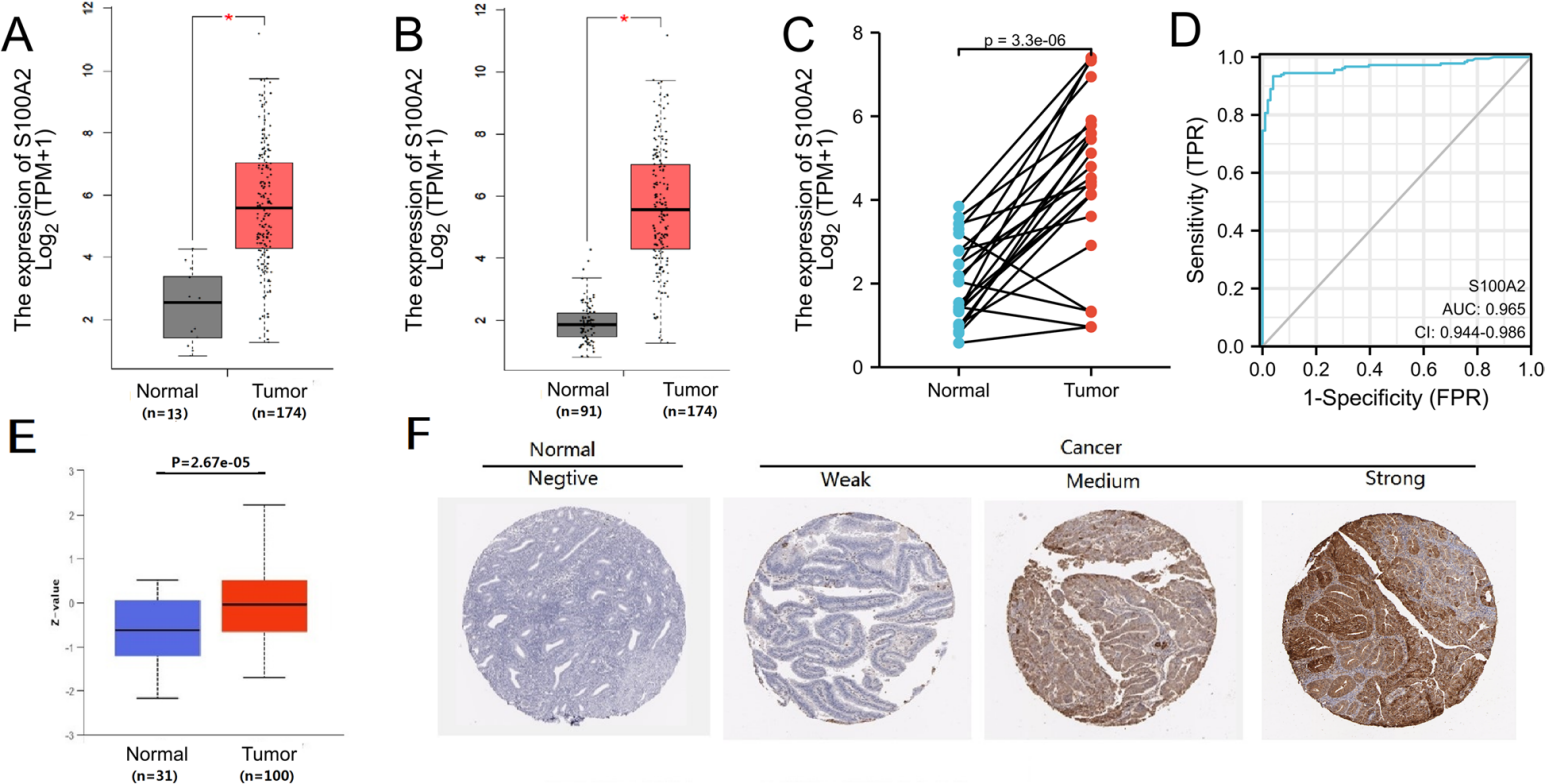
High S100A2 expression is correlated with clinicopathologic features in patients with endometrial carcinoma. (**a**) Differences in S100A2 expression in UCEC tissues and adjacent normal tissues. (**b**) Differences in S100A2 expression in normal samples from the GTEx combined adjacent UCEC tissues and UCEC samples. (**c**) Differences in S100A2 expression in UCEC samples and paired adjacent samples. (**d**) ROC curve for S100A2 in normal samples from the GTEx combined adjacent UCEC tissues and UCEC samples. (**e**) S100A2 protein levels were markedly upregulated in tumor tissues compared with that in non-paired normal tissues. (**f**) Representative images of S100A2 expression in endometrial carcinoma tissues and their normal controls

*S100A2* was found high expressed in the GSE17025 and GSE39099 profiles and screened out among the six genes. To examine the role of *S100A2* in endometrial carcinoma progression, TCGA databases were used to predict the *S100A2* mRNA expression patterns in 174 endometrial carcinoma tissue samples and 13 normal tissue samples (Fig. 2a). The results showed that *S100A2* mRNA expression levels were significantly higher in endometrial carcinoma primary tumor samples than in normal tissue (*P* < 0.05). We further compared the expression of *S100A2* in normal samples of GTEx combined adjacent UCEC tissues and UCEC samples, and found that *S100A2* was overexpressed in UCEC (*P* < 0.05) (Fig. 2b). Additionally, *S100A2* expression was significantly upregulated in 23 endometrial carcinoma samples when compared with that in matched adjacent samples (*P* = 3.3e−06) (Fig. 2c). We also plotted a receiver operating characteristic (ROC) curve to evaluate the diagnostic value of *S100A2* levels by comparing *S100A2* expression in normal samples of GTEx combined adjacent UCEC tissues and UCEC samples. The area under the curve (AUC) value for *S100A2* levels was found to be 0.965 (CI = 0.944*–*0.986), suggestive of a high diagnostic potential (Fig. 2d). The protein expression level of S100A2 was also upregulated in endometrial carcinoma tissues in comparison with normal tissues (Fig. 2e and f), indicating that the protein and mRNA expressions of S100A2 were similar in different database.

The gene-gene and protein-protein interaction network, which was generated by using GeneMANIA and STRING showed that 20 potential target genes and 10 potential target proteins interacted with the S100A2 (Fig. S1).

### Correlation between S100A2 expression and clinical characteristics

**Fig. 3:**
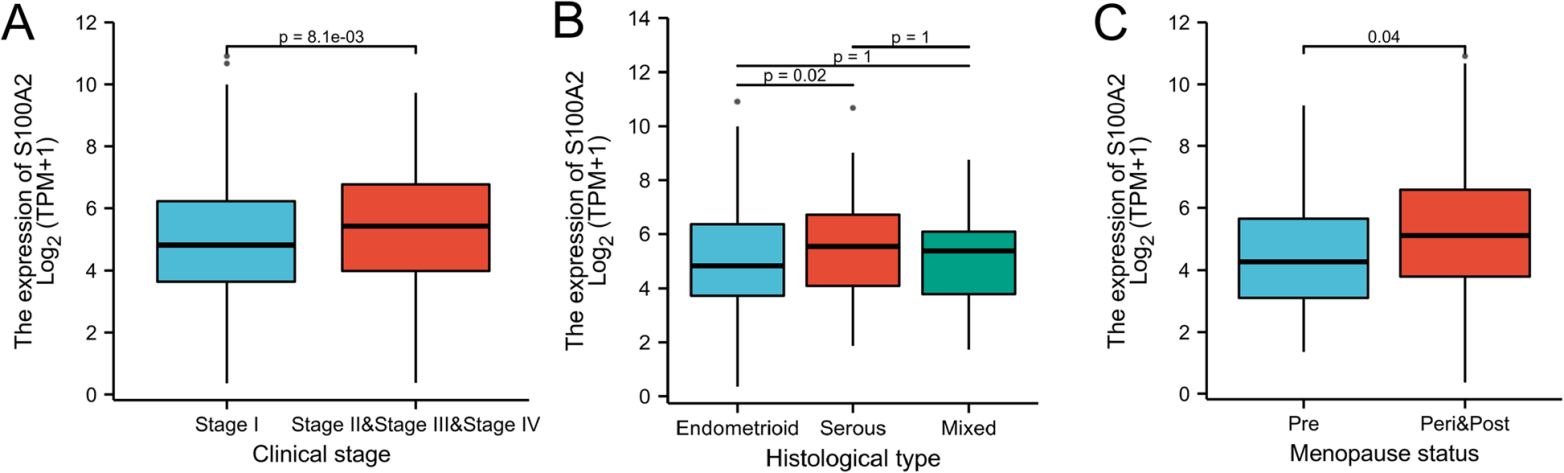
Box plot assessing S100A2 expression in patients with endometrial carcinoma according to different clinical characteristics. (**a**) Stage, (**b**) histological type, and (**c**) menopause status

The characteristics of 552 patients with endometrial carcinoma, including clinical and gene expression data, were collected from TCGA database. The patients were divided into high and low S100A2 expression groups based on the mean value of S100A2 expression (Table 1), following which putative correlations between S100A2 expression and clinical characteristics were evaluated using the Wilcoxon signed-rank test and logistic regression analysis. The results showed that S100A2 mRNA expression differed significantly between stage I and stage II-IV tumors (*P* = 8.1e−03) as well as between menopause status (pre-menopause vs. peri- and post-menopause, *P* = 0.04) (Fig. 3a and c). S100A2 expression was also upregulated in serous type of endometrial carcinoma compared with that in endometrioid type disease (*P* = 0.02) (Fig. 3b).

**Table 1:** The relationship between S100A2 mRNA expression and clinical characteristics in endometrial carcinoma

Characteristic	Low expression of S100A2	High expression of S100A2	*P* value
n	276	276	
Clinical stage, n (%)			**0.028**
Stage I	186 (33.7%)	156 (28.3%)	
Stage II	22 (4%)	29 (5.3%)	
Stage III	52 (9.4%)	78 (14.1%)	
Stage IV	16 (2.9%)	13 (2.4%)	
Primary therapy outcome, n (%)			0.864
PD	11 (2.3%)	9 (1.9%)	
SD	2 (0.4%)	4 (0.8%)	
PR	6 (1.2%)	6 (1.2%)	
CR	218 (45.4%)	224 (46.7%)	
Race, n (%)			0.549
Asian	10 (2%)	10 (2%)	
Black or African American	60 (11.8%)	48 (9.5%)	
White	188 (37.1%)	191 (37.7%)	
Age, n (%)			0.766
<=60	101 (18.4%)	105 (19.1%)	
>60	174 (31.7%)	169 (30.8%)	
Weight, n (%)			0.727
<=80	124 (23.5%)	119 (22.5%)	
>80	140 (26.5%)	145 (27.5%)	
BMI, n (%)			0.219
<=30	114 (22%)	98 (18.9%)	
>30	147 (28.3%)	160 (30.8%)	
Histological type, n (%)			**0.014**
Endometrioid	220 (39.9%)	190 (34.4%)	
Mixed	9 (1.6%)	15 (2.7%)	
Serous	47 (8.5%)	71 (12.9%)	
Residual tumor, n (%)			0.962
R0	198 (47.9%)	177 (42.9%)	
R1	12 (2.9%)	10 (2.4%)	
R2	8 (1.9%)	8 (1.9%)	
Histologic grade, n (%)			0.658
G1	51 (9.4%)	47 (8.7%)	
G2	63 (11.6%)	57 (10.5%)	
G3	156 (28.8%)	167 (30.9%)	
Tumor invasion(%), n (%)			0.402
<50	134 (28.3%)	125 (26.4%)	
>=50	102 (21.5%)	113 (23.8%)	
Menopause status, n (%)			**0.038**
Pre	23 (4.5%)	12 (2.4%)	
Peri	5 (1%)	12 (2.4%)	
Post	221 (43.7%)	233 (46%)	
Hormones therapy, n (%)			0.578
No	143 (41.6%)	154 (44.8%)	
Yes	20 (5.8%)	27 (7.8%)	
Radiation therapy, n (%)			0.177
No	148 (28.1%)	131 (24.9%)	
Yes	116 (22%)	132 (25%)	
Diabetes, n (%)			0.158
No	155 (34.4%)	173 (38.4%)	
Yes	68 (15.1%)	55 (12.2%)	

Univariate logistic regression analysis demonstrated that *S100A2* expression was correlated with some clinical characteristics in patients with endometrial carcinoma (Table 2). A comparison of baseline data between the high and low expression groups revealed that *S100A2* expression was significantly associated with clinical stage (odds ratio [OR] = 1.505, *P* = 0.031), histological type (mixed and serous vs. endometrioid: OR = 1.778, *P* = 0.004), and menopause status (OR = 2.078, *P* = 0.047).

**Table 2:** S100A2 expression associated with clinicopathologic characteristics (logistic regression)

Characteristics	Total(N)	Odds Ratio(OR)	*P* value
Clinical stage (Stage IV&Stage III vs. Stage I&Stage II)	552	1.505 (1.039-2.186)	**0.031**
Primary therapy outcome (PD&SD&PR vs. CR)	480	0.973 (0.499-1.897)	0.936
Weight (>80 vs. <=80)	528	1.079 (0.766-1.520)	0.662
Age (>60 vs. <=60)	549	0.934 (0.661-1.320)	0.700
BMI (>30 vs. <=30)	519	1.266 (0.892-1.800)	0.187
Histological type (Serous&Mix vs. Endometrioid)	552	1.778 (1.209-2.632)	**0.004**
Histologic grade (G3 vs. G1&G2)	541	1.173 (0.832-1.656)	0.362
Residual tumor (R1&R2 vs. R0)	413	1.007 (0.512-1.967)	0.984
Tumor invasion(%) (>=50 vs. <50)	474	1.188 (0.827-1.707)	0.352
Menopause status (Peri&Post vs. Pre)	506	2.078 (1.028-4.408)	**0.047**
Diabetes (Yes vs. No)	451	0.725 (0.477-1.098)	0.129

### The independent diagnostic value of S100A2 expression in endometrial carcinoma

**Fig. 4:**
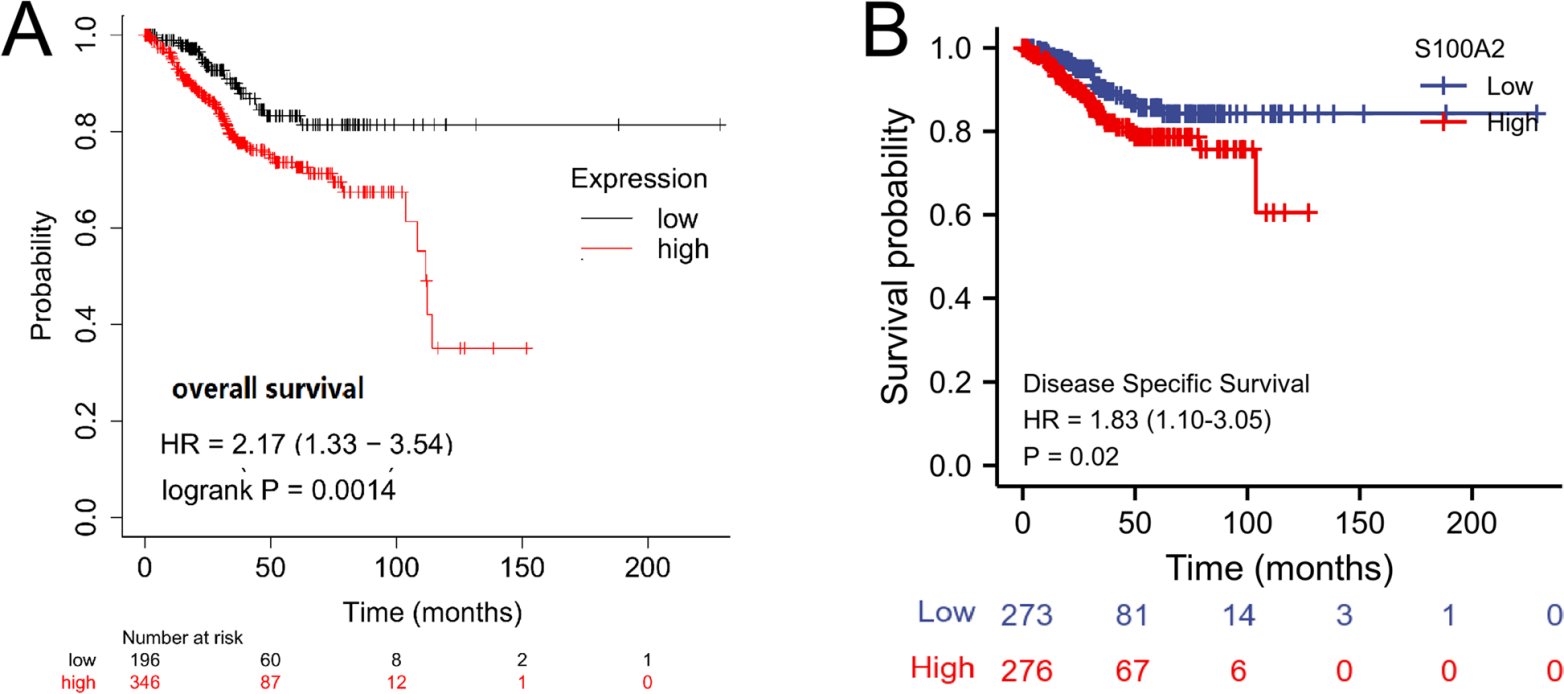
The independent risk and diagnostic value of S100A2 expression in endometrial carcinoma. (**a** & **b**) Kaplan–Meier analysis of overall survival (OS) and disease specific survival (DSS) for patients

**Fig. 5:**
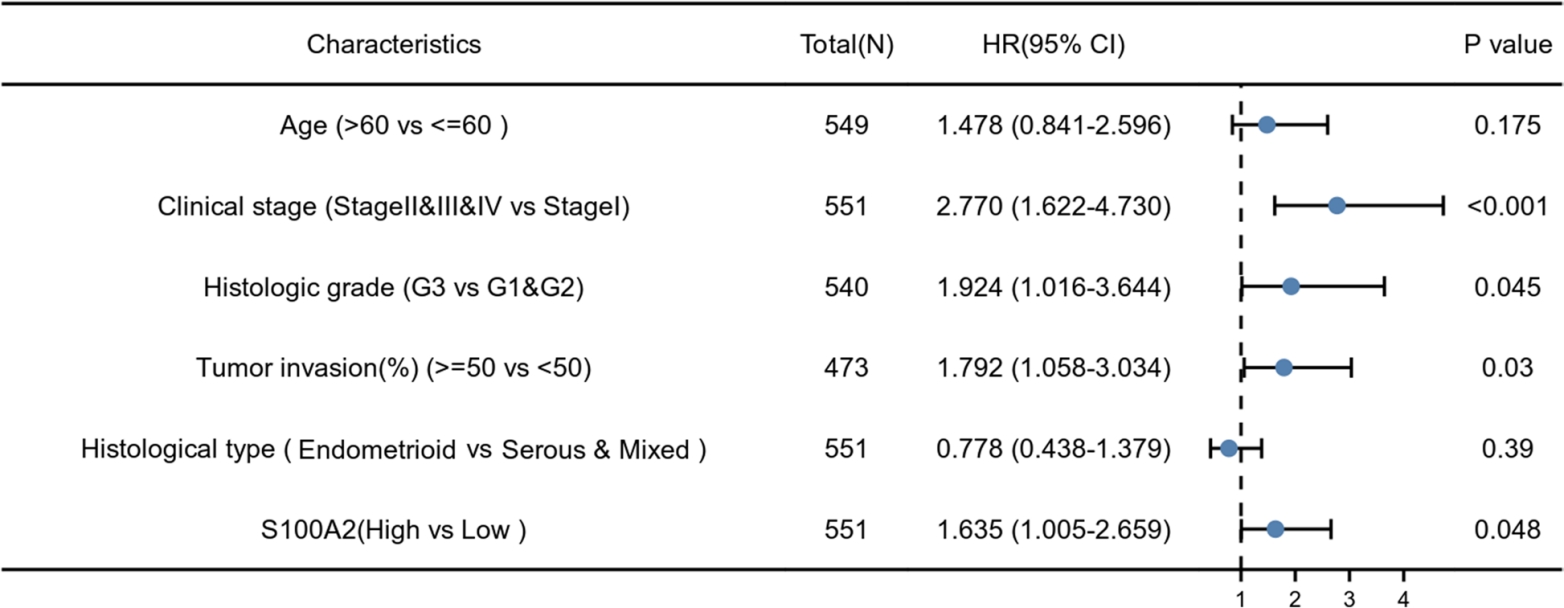
Forest plot of the multivariate Cox regression analysis in endometrial carcinoma patients

**Fig. 6:**
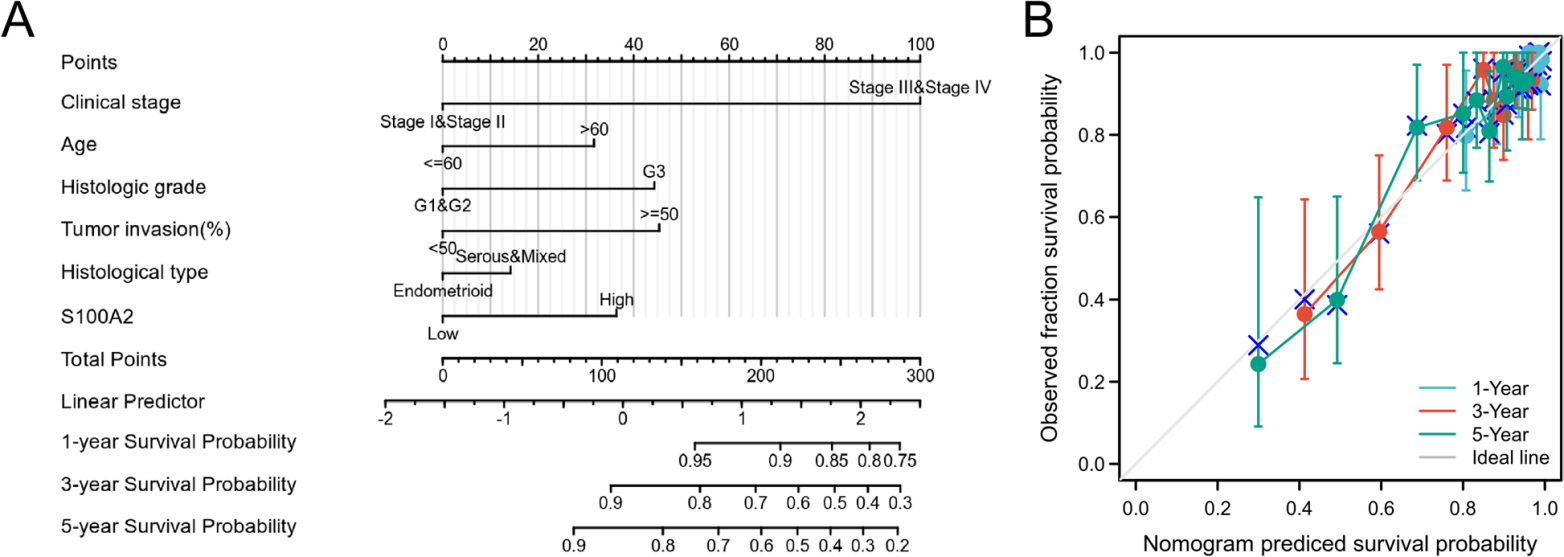
Construction and validation of nomogram based on S100A2 expression. (**a**) A nomogram for predicting the probability of 1-, 3- and 5-year OS in endometrial carcinoma patients. (**b**) Calibration plots validating the efficiency of nomograms for OS

Survival analysis demonstrated that high S100A2 expression was correlated with poor OS (*P* = 1.4e−03) as well as poor DSS (*P* = 0.02) (Fig. 4a and b). Univariate Cox regression analysis showed that high S100A2 expression was significantly correlated with poor OS (HR = 1.616, 95% CI = 1.069-2.442). Moreover, multivariate regression analysis further confirmed that S100A2 expression was an independent prognostic factor for OS in patients with endometrial carcinoma (HR = 1.635, 95% CI = 1.005-2.659, *P* = 0.048) (Table 3; Fig. 5). Subsequently, a nomogram was constructed using age, clinical stage, histologic grade, tumor invasion, histological type and S100A2 levels as indicators to predict 1-, 3-, and 5-year OS in patients with endometrial carcinoma (Fig. 6a). The calibration curve presented desirable prediction of the nomograms for the 1-, 3-, and 5-year clinical outcomes (Fig. 6b). Combined, the above data indicated that S100A2 may serve as a useful biomarker for the prediction of OS among endometrial carcinoma patients.

**Table 3:** Correlations between overall survival and S100A2 mRNA expression using univariate and multivariate Cox regression

Characteristics	Total (N)	Univariate analysis		Multivariate analysis	
		Hazard ratio (95% CI)	P value	Hazard ratio (95% CI)	*P* value
Age (>60 VS <=60)	549	1.847 (1.160-2.940)	**0.010**	1.478 (0.841-2.596)	0.175
Clinical stage (Stage IV&Stage III&Stage II VS Stage I)	551	3.270 (2.145-4.984)	**<0.001**	2.770 (1.622-4.730)	**<0.001**
Histologic grade (G3 VS G2&G1)	540	3.281 (1.907-5.643)	**<0.001**	1.924 (1.016-3.644)	**0.045**
Tumor invasion(%)(>=50 VS <50)	473	2.813 (1.744-4.535)	**<0.001**	1.792 (1.058-3.034)	**0.030**
BMI(>30 VS <=30)	518	1.034 (0.680-1.572)	0.876		
Menopause status (Peri&Post VS Pre)	505	0.828 (0.382-1.794)	0.632		
Histological type (Endometrioid VS Serous & Mixed)	551	0.380 (0.253-0.573)	**<0.001**	0.778 (0.438-1.379)	0.390
S100A2 (High VS Low)	551	1.616 (1.069-2.442)	**0.023**	1.635 (1.005-2.659)	**0.048**

### S100A2-related signaling pathways based on GSEA

**Fig. 7:**
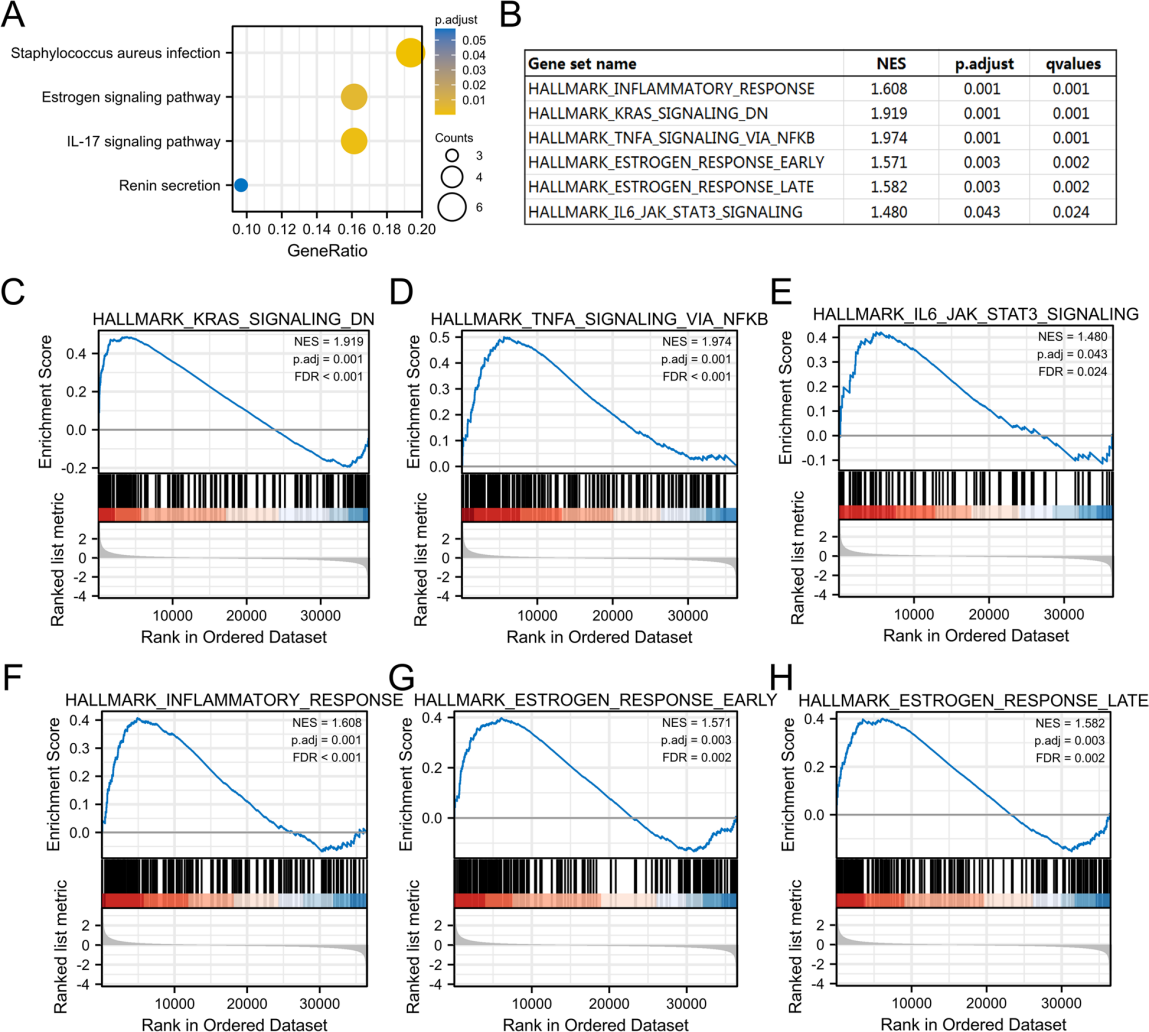
Functional enrichment analysis of S100A2 in endometrial carcinoma. (**a**) KEGG pathway analysis. **(b**) Enrichment plots of S100A2-relevant enrichment pathways in h.all.v7.2.symbols.gmt from GSEA. (**c**) **c** KRas-signaling-DN, (**d**) TNFα-signaling-via-NF-κB, (**e**) IL6-JAK-STAT3-signaling (**f**) Inflammatory response (**g**) estrogen-response-early and (**h**) estrogen-response-late

Next, we sought to identify the putative cellular mechanisms underlying the effects of S100A2 in endometrial carcinoma through KEGG pathway analysis and GSEA. As shown in Fig. 7a, enrichment analysis indicated that hsa05150 (*Staphylococcus aureus* infection) was the pathway most strongly associated with the high S100A2 expression group, while hsa04657 (IL-17 signaling pathway) and hsa04915 (estrogen signaling pathway) were also found to be associated with the role of S100A2 in endometrial carcinoma. Meanwhile, inflammatory response, estrogen response, JAK/STAT3, K-Ras, and TNFα/NF-κB were the most differentially enriched pathways in S100A2 high expression phenotype (Fig. 7b–h).

## Discussion

Some members of the S100 family have been identified as playing a tumorigenic role in endometrial carcinoma. For instance, S100A4/non-muscle myosin II signaling has been associated with epithelial*–*mesenchymal transition and stemness in uterine carcinosarcoma [18], while the inhibition of *S100A8* expression is reported to promote apoptosis through the suppression of AKT phosphorylation [19]. Although there have been studies linking S100A8, S100A11 as biomarker candidates for endometrial cancer detection [20], whether any other S100 gene family member is involved in endometrial carcinoma remains unclear. Accordingly, in this study, we analyzed the expression and prognostic value of different *S100* genes in this cancer. High S100A2 expression was previously reported to be an independent prognostic biomarker for poor prognosis in stage II and III colorectal cancer [21]. Here, we found that S100A2 also has prognostic significance for endometrial carcinoma.

Different from other S100 proteins, S100A2 is mainly localized to the nucleus and exerts its biological role in a tissue- and cell-specific manner [22]. The expression of several S100 family members is dysregulated in multiple types of tumors, including pancreatic, head and neck, bladder, and prostate cancers [23–26]. Until recently, S100A2 was thought to be the only protein in the S100 family that was negatively correlated with tumor initiation and progression; however, it was recently shown that high S100A2 expression is associated with FIGO stage as well as with histologic subgroups in gastric cancer [27]. High cytoplasmic S100A2 and/or total S100A2 expression levels were identified as predictors of recurrence risk in patients with oral cancer [28]. High expression of S100A2 is also significantly correlated with worse OS in patients with ovarian cancer [29]. Consistent with these observations, our results revealed that, compared with normal tissue, S100A2 expression was significantly upregulated in endometrial carcinoma tissues at both the mRNA and protein levels.

We further investigated the prognostic value of S100A2 in endometrial carcinoma using Kaplan–Meier plotter. The results showed that patients with high *S100A2* mRNA expression levels had worse OS and DSS, while multivariate Cox analysis confirmed that high S100A2 expression was an independent risk factor for OS in individuals with endometrial carcinoma. ROC analysis also confirmed the diagnostic value of S100A2. According to the FIGO 2009 staging systems, the 5-year survival rate for patients with endometrial carcinoma was 89.6 ~ 77.6% for stage I and 57.0 ~ 49.4% for stage III disease [30]. A recent study further reported a predictive model for OS consisting of age, clinical stage, pathological tissue grade, tumor size, and ethnicity [31]. A predictive nomogram for endometrial carcinoma combining long noncoding RNA expression profiles and m6A regulator-related CpG sites was also shown recently [32, 33]. Here, we constructed a new prognostic nomogram using age, clinical stage, histologic grade, tumor invasion, histological type and S100A2 levels as indicators that can be used by physicians to improve the accuracy of identifying high-risk patients. We further investigated the relationship between clinical characteristics and *S100A2* mRNA expression in endometrial carcinoma patients and found that high S100A2 expression was associated with clinical stage and menopause status. Furthermore, our results demonstrated that the associated mechanisms may involve the inflammatory response, K-Ras signaling, TNFα signaling *via* NF-kB, the IL6/JAK/STAT3 axis, and estrogen response. Although increased levels of estrogens in the blood are believed to encourage the development of endometrial cancer [34], the crosstalk between estrogen and S100A2 remains poorly understood. Collectively, these findings demonstrated that S100A2 may be a promising biomarker for the diagnosis of endometrial carcinoma.

High S100A2 expression has been associated with the regulation of tumor cell proliferation and invasion through the enhancement of PI3K/AKT activation and functional interaction with SMAD3 [35]; however, whether S100A2 expression has prognostic value in endometrial carcinoma has remained unknown. S100A2 is thought to act both as a tumor suppressor and as an oncogene depending on the tumor type, suggesting that S100A2 may exert its effects through different mechanisms. GSEA analysis revealed that S100A2 interacts with the IL-17 signaling pathway in endometrial carcinoma. The IL-17 signaling cascade is thought to promote cytokine and chemokine production. After binding to its receptor, IL-17 activates mitogen-activated protein kinases, leading to the production of inflammatory mediators such as IL-6, IL-1, and NF-κB. It is well known that IL-17 acts both directly and indirectly on tumor cells, leading to tumor microenvironment remodeling. Similar to low-grade glioma, in which the S100A family genes play vital roles in tumor progression mostly via the IL-17 signaling pathway [36], our data implied that S100A2 might play a critical role in endometrial carcinoma initiation and progression through similar mechanisms. Although the current study revealed a potential interaction between S100A2 and the IL-17 signaling pathway, how S100A2 precisely regulates endometrial carcinoma progression requires further investigation.

## Conclusions

In conclusion, this analysis revealed that S100A2 was more highly expressed in endometrial carcinoma tissues than in normal tissues and was correlated with worse survival. S100A2 has potential as a prognostic factor for patients with endometrial carcinoma. Recently, an inhibitor of S100A9 was used in clinical trials as a therapy for prostate cancer and other solid tumors [37]. Our study provides new insights regarding the contribution of S100A2 to endometrial carcinoma progression and may be of empirical value for future investigations on inhibitors targeting S100A2 for the treatment of endometrial carcinoma.

## Supplementary Information

**Additional file 1.**

## Data Availability

The datasets supporting the conclusions of this article are included within the article.

## Abbreviations

CI: Confidence Interval
DEGs: Differentially Expressed Genes
EGA: European Genome-phenome Archive
GEPIA: Gene Expression Profiling Interactive Analysis
GTEx: Genotype-Tissue Expression
GEO: Gene Expression Omnibus
GSEA: Gene Set Enrichment Analysis
KEGG: Kyoto Encyclopedia of Genes and Genomes
HR: Hazard Ratio
NCBI: National Center for Biotechnology Information
NES: Normalized Enrichment Score
OR: Odds Ratio
OS: Overall Survival
DSS: Disease Specific Survival
ROC: Receiver Operating Characteristic
TCGA: The Cancer Genome Atlas
UCEC: Uterine Corpus Endometrial Carcinoma
