# Supplementary material for: High expression of S100A2 predicts poor prognosis in patients with endometrial carcinoma
Source: BMC Cancer. 2022 Jan 18;22:77. doi: 10.1186/s12885-022-09180-5 (PMC8764844; doi:10.1186/s12885-022-09180-5)
Supplement: Supplementary file 1 — Additional file 1. [file 12885_2022_9180_MOESM1_ESM.docx]

**
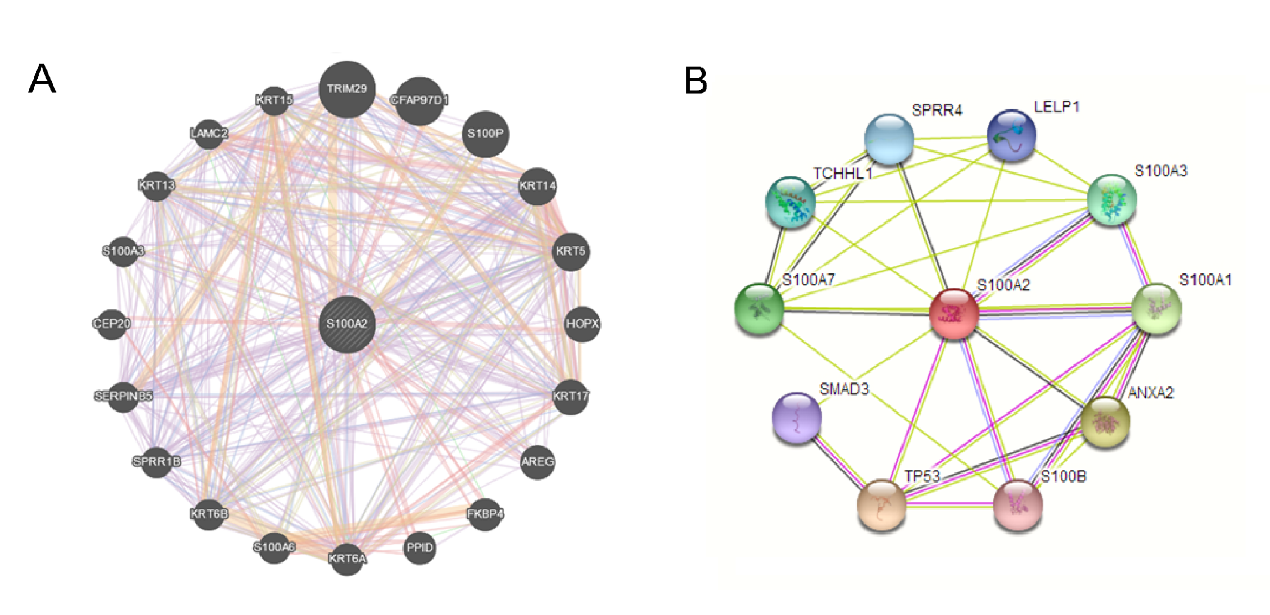
**

**Fig. S1 Gene-gene and protein-protein interaction network of S100A2.**

(a) The gene network associated with the S100A2 drawn by using GeneMANIA. (b) A network diagram of interactions between proteins encoded S100A2, drawn by using STRING.
